# Identifying the origin of atrial tachycardia in the epicardial region by analyzing two separate roving activation intervals using a novel three-dimensional mapping system: A case study

**DOI:** 10.1016/j.hrcr.2022.05.024

**Published:** 2022-06-03

**Authors:** Takashi Kanda, Masaharu Masuda, Naoya Kurata, Yusuke Katagiri, Yasuhiro Matsuda, Toshiaki Mano

**Affiliations:** ∗ Department of Cardiology, Osaka Police Hospital, Osaka, Japan; † Kansai Rosai Hospital Cardiovascular Center, Amagasaki, Japan

**Keywords:** Atrial tachycardia, High-density cardiac mapping, Vein of Marshall, Ethanol infusion, Novel mapping system

**Key Teaching Points**

• Marshall bundle–related atrial tachycardias (AT) are common after atrial fibrillation ablation, but their identification with conventional 3-dimensional mapping techniques may be difficult.
• In analyzing the tachycardia mechanism, omnipolar technology, grid mapping catheter, and the improvement of noise filtering contributed to the observation of epicardial potentials.
• In addition to the high-resolution performance of the EnSite X EP System (Abbott, St. Paul, MN), it was useful in assessing tachycardia through the evaluation of propagation with an annotation-dependent activation map and annotation-independent omnipolar vectors, and changing the roving activation intervals as needed for circuit identification.
• Vein of Marshall (VOM) ethanol infusion is a feasible and safe treatment for VOM-related AT.

## Introduction

Atrial tachycardias (ATs) after atrial fibrillation (AF) ablation usually require electroanatomic mapping to obtain a precise diagnosis and guide ablation. The Marshall bundle (MB), which is often involved in the occurrence of AT, is a myocardial sleeve contained in the ligament of Marshall together with the vein of Marshall (VOM) and autonomic nerves in the epicardial aspect of the left lateral ridge.^1^, ^2^, ^3^ This muscle structure has been shown to be capable of generating focal automatic activity. However, epicardial connections may impede interpretation of the endocardial activation sequence and the efficacy of radiofrequency ablation.^4^^,^^5^

Here, we present a case in which we used a novel mapping system to identify the origin of a post–AF ablation AT related to the VOM.

## Case report

**Figure 1:**
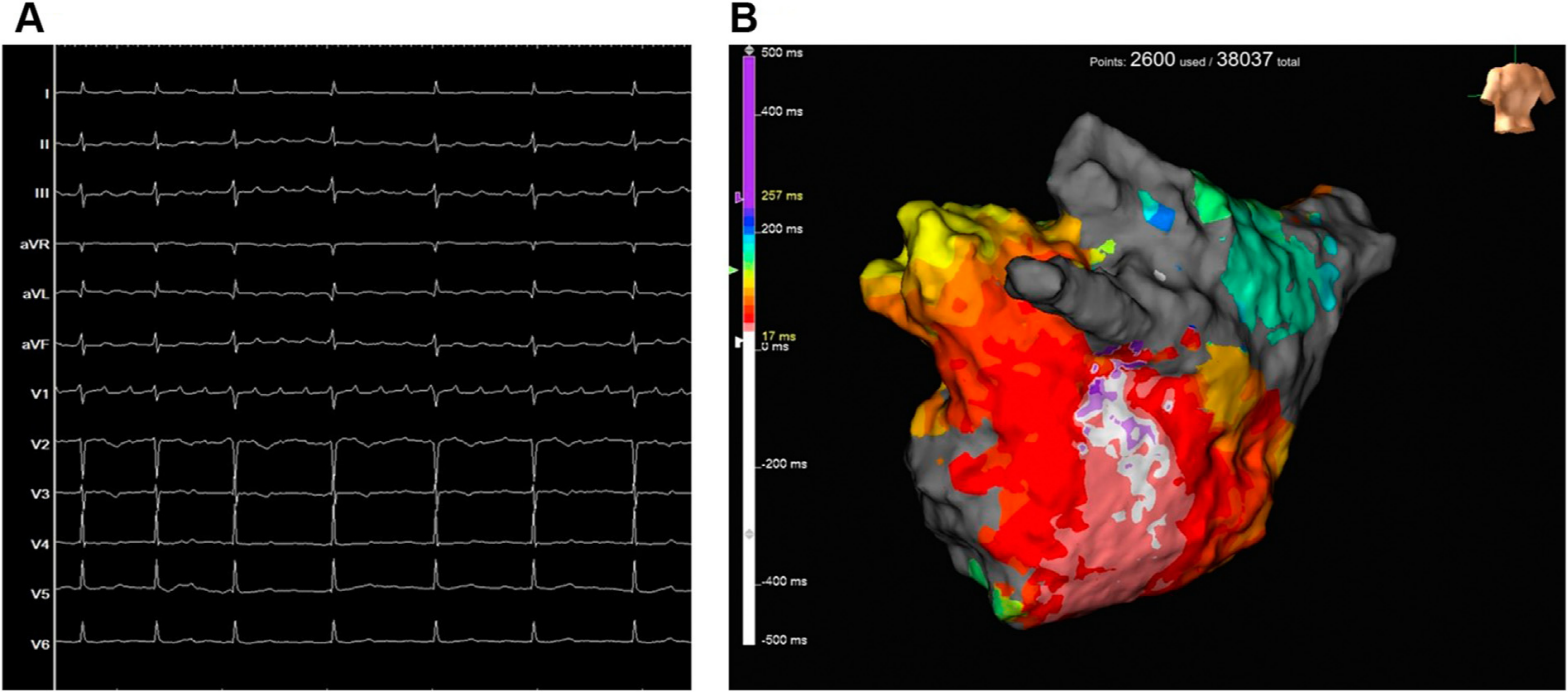
**A:** Twelve-lead electrocardiogram during atrial tachycardia. **B:** Left atrial conventional activation map during atrial tachycardia. The conventional activation map identified the earliest excitation site in a wide area downward from the bottom of the left inferior pulmonary vein.

The patient was a 76-year-old woman with a history of AF ablation undertaken 7 years (pulmonary vein isolation) and 5 years (repeat pulmonary vein isolation and superior vena cava isolation) previously who was admitted for a recurrent AT (98 beats/min) with mild exertional dyspnea and palpitation (Figure 1A). In the 2 previous ablation procedures, the only ablation site for the left atrium was the pulmonary vein.

During an episode of AT with a cycle length of 250 ms, endocardial mapping was performed using the Advisor HD Grid Mapping Catheter (GMC; Abbott, St. Paul, MN). The mapping system (EnSite X EP System) includes a recording device (HD Wave Solution®) that can record 32 bipolar signals (16 along the splines and 16 across the splines) and novel omnipolar technology (OT). In this case, excitation propagation of the catheter placed in the coronary vein was distal to proximal, and the AT was considered to be of left atrial origin. Entrainment pacing was performed at coronary sinus (CS) distal, CS proximal, left atrial lateral wall, and right atrial, and postpacing interval and tachycardia cycle length were consistent at CS distal.

**Figure 2:**
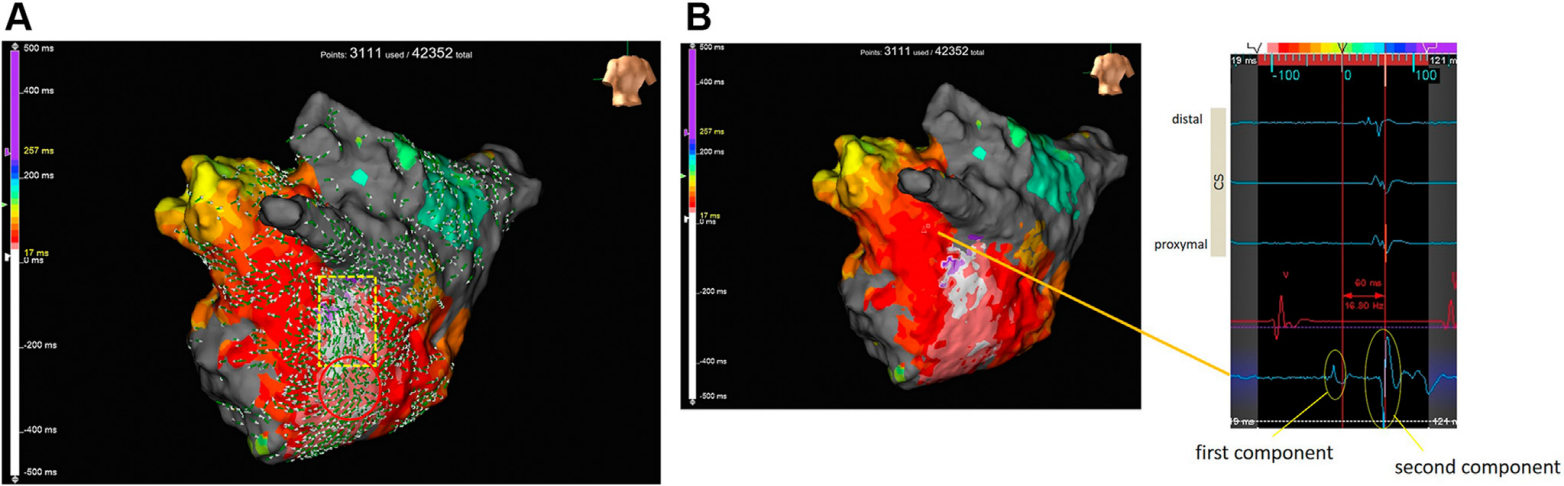
**A:** Left atrial activation map with omnipolar vectors. The yellow dotted line shows vectors flowing from upward to downward, and the red circle shows a breakout of excitement around the area. **B:** Analysis of local potentials showed that they were composed of 2 components: point A was annotated to a delayed potential.

From these findings, we made a map of the left atrium using GMC. The conventional activation map suggested a focal AT pattern from the lower left inferior pulmonary vein (LIPV) (Figure 1B). However, the map using omnipolar potential showed arrows from the LIPV to the origin of the focal AT, suggesting the presence of some electrical conduction upstream of the breakout site of focal AT (Figure 2A).

**Figure 3:**
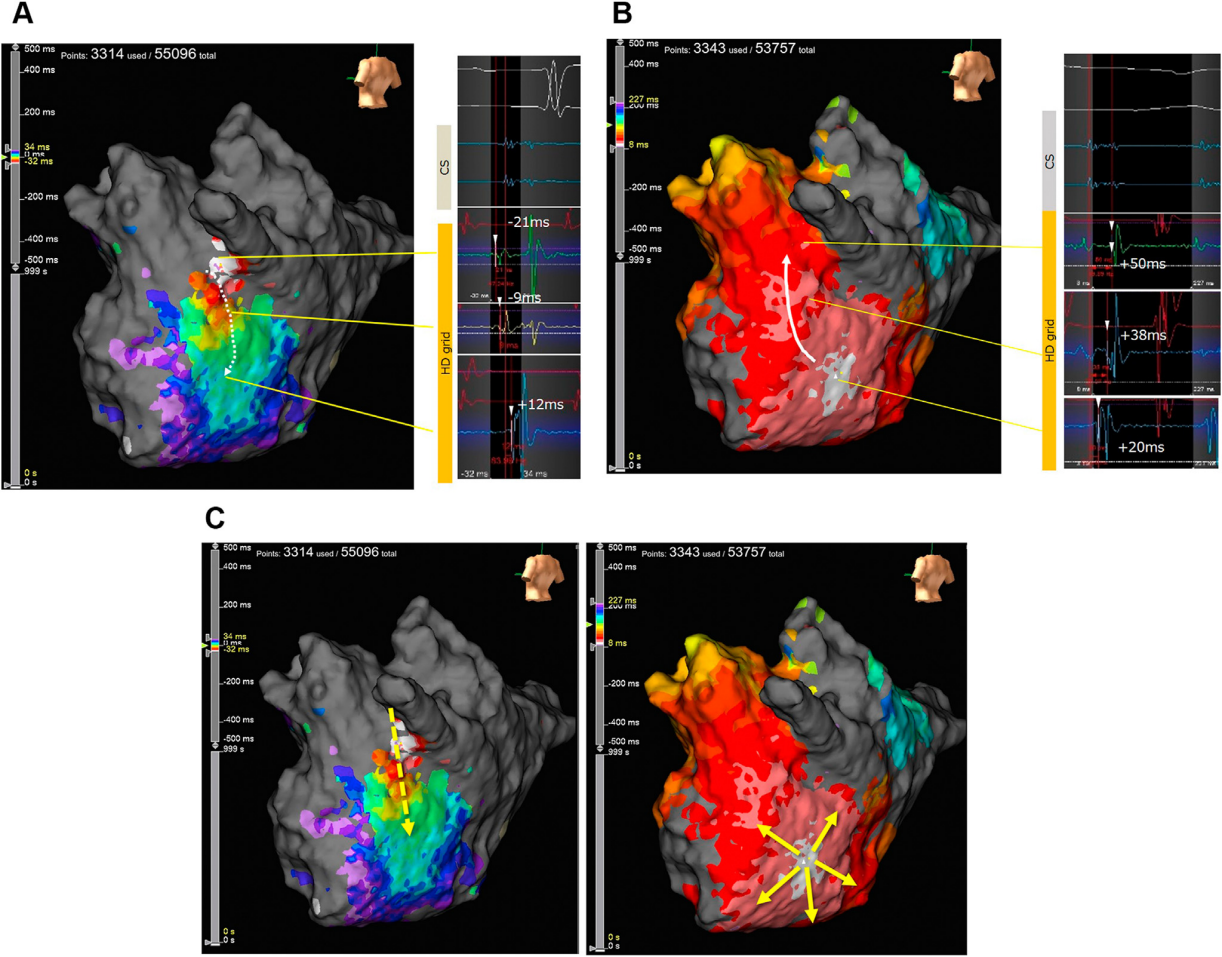
Activation map created by dividing the roving activation interval into 2 segments. **A:** Map focused on the first component shows a downward propagation of excitation from the left pulmonary vein–left atrial appendage (LAA) ridge. **B:** Map focused on the second component shows a centrifugal pattern occurring downward of the left inferior pulmonary vein (LIPV), as in the conventional activation map (Figure 1B). **C:** Illustration of a tachycardia circuit. Epicardial conduction descended from the LAA ridge from the LIPV anterior to the LIPV inferior. Endocardial conduction spread in concentric circles in the left atrium from the LIPV inferior.

In the acquired map, local potentials around the LIPV were examined in detail and were found to be composed of 2 components (Figure 2B). We therefore divided the window (roving activation interval) into the 2 components and recreated the activation map. The map focused on the first component showed a downward propagation of excitation from the left pulmonary vein–left atrial appendage (LAA) ridge (Figure 3A), while the map focused on the second component showed a centrifugal pattern downward from the LIPV, as in the conventional map described above (Figure 3B). In addition, when entrainment pacing was performed near the junction of the VOM and coronary vein, the postpacing interval was consistent with the tachycardia cycle length. However, the tachycardia was not terminated by radiofrequency ablation using an ablation catheter near the junction of the VOM and coronary vein or from the endocardial side on the contralateral side of the same site. This finding suggests that the mechanism of AT is a reentrant tachycardia involving epicardial circuits.

These results suggest that the VOM-related epicardial conduction, descending from the LAA ridge from the left pulmonary vein anterior to the left pulmonary vein inferior, broke out to the left atrial endocardium at the LIPV inferior as the mechanism of AT (Figure 3C). Since the VOM was running near the site where the potential of the first component (presumably the VOM potential) was recorded, ethanol injection was planned. The CS was cannulated with a steerable long sheath (Agilis NxT; Abbott, St Paul, MN) inserted from the right femoral vein. An angiography catheter (5F left internal mammary artery; Medtronic, Minneapolis, MN) was proximally positioned inside the CS lumen. An angioplasty wire (Cruise 0.014; ASAHI Intecc, Aichi, Japan) was then advanced inside the VOM lumen and used to position a preloaded over-the-wire balloon (Emerge, 1.5 mm diameter and 8 mm length; Boston Scientific, Natick, MA) within the first 15 mm of its proximal portion. After inflation at 6 atm and wire removal, selective angiography was performed through the wire port to confirm balloon occlusion and visualize VOM arborization. AT was terminated about 2 seconds after injection of 2 mL ethanol 98% (Supplemental Figure 1). At the end of the procedure, lack of inducibility was accepted as an indicator of successful ablation. Six months post ablation, the patient had no recurrence of arrhythmia and remained asymptomatic without antiarrhythmic treatment.

## Discussion

In this case, we used the EnSite X EP System to treat the patient’s AT. The OT allowed us to determine and treat the excitation propagation through the VOM by evaluating the vectors, which were difficult to identify with conventional mapping. To our knowledge, this is the first report of treatment with the EnSite X EP System for VOM-related AT. By dividing the tachycardia cycle into the 2 windows and creating maps based on the findings of OT, we were able to visualize this tachycardia circuit involving the VOM.

### Detailed analysis of tachycardia with a novel mapping system

A variety of 3-dimensional (3D) mapping systems are currently available, each of which is capable of acquiring high-density maps using a multipolar catheter. Using the EnSite X EP System and GMC in this case, we were able to record the epicardial side of the potential, which enabled us to understand the entire circuit of the patient’s AT.

In analyzing the tachycardia mechanism in this case, we believe that OT, GMC, and improvements in noise filtering contributed to the observation of epicardial potentials. OT in EnSite X uses both unipolar and bipolar signals recorded with GMC to obtain omnipolar signals, directions, and speeds. OT facilitates diagnosis and treatment decisions by providing a new way to calculate bipolar electrograms that are independent from catheter-wavefront orientation.^6^ GMC allows sufficient contact between the catheter and tissue, contributing to a secure endocardial contact and in turn allowing the detailed recording of electrical potential.^7^ The EnSite X EP System reduces noise compared to previous systems, making it possible to analyze extremely small electrical potentials.

Regarding the involvement of the MB, we believe that sufficient evidence for this is found in the findings of 3D mapping described above, and in the facts that electrograms other than those around the VOM showed almost no scarring on the map after ethanol injection and that the tachycardia terminated without any widespread effect of ethanol.

### Differences between the EnSite X EP System and conventional mapping systems

As shown in Figure 2A, we found omnipolar vectors with different orientation from the conventional activation map, which allowed us to evaluate local potentials in detail.

In the activation map (Figure 1) obtained using the conventional method, the annotation setting was set to detect -dV/dt, and when there were 2 components, 1 of the components was canceled. If the intracardiac local potential has multiple components, it may not be possible to visualize the true arrhythmia circuit without changing the settings. In contrast, when we set up a window for each of the 2 potentials, the propagation maps showed that the 2 were excited in opposite directions. Since 1 of the potentials was small and showed excitation consistent with MB anatomy, we considered it to be excitation of the MB on the epicardial side.

By dividing the tachycardia cycle into the 2 parts, we were able to visualize conduction through the MB and conduction on the endocardial side (Figure 3). We also obtained an interesting finding when we used the omnipolar vector to visualize tachycardia using the same method of dividing the window.

Some of the omnipolar vectors did not change their orientation even when the window was divided into the 2 components. The activation map including the first signal component showed excitation from near the LAA to the lower part of the LIPV, as in the conventional map. In contrast, in the activation map including the second signal component, even though the excitation spread from the lower part of the LIPV, the direction of the arrow was displayed in the opposite direction and did not change from that in the first signal component map (Supplemental Figure 2). Although details of the mechanism have not been disclosed, OT has a mechanism that evaluates potentials between approximately 60 ms before and after the annotated time phase (subinterval), even if they are outside the roving activation interval. This feature may explain how the above phenomenon occurred.

### Treatment of VOM-related AT

Connections of the VOM to the adjacent myocardium vary. Vlachos and colleagues^5^ reported that VOM-related ATs accounted for up to 30.2% of the left ATs after AF ablation. They report that about half of the VOM-related ATs were macroreentry ATs and the other half were localized reentry. Regarding treatment, VOM-related ATs can be terminated with radiofrequency ablation, either endocardially via VOM–left atrium connection or epicardially via VOM-CS connections (at the ostium of the VOM), and with ethanol infusion inside the VOM.^5^ The results of VOM ethanol infusion were reported by Kamakura and colleagues^8^ based on more than 700 patients treated, with an efficacy of 88.9% and serious complications in only 2% of patients. Generally, MB-related ATs described to date have mainly occurred after extensive substrate ablation, and reports after PV isolation only are rare.^9^ However, Margato and colleagues^4^ detailed a similar case to ours using the Rhythmia system (Boston Scientific), in which they were able to diagnose and treat AT of the MB-mediated circuit using the annotation-independent LUMIPOINT software with a high-resolution mapping system. The LUMIPOINT software can be used for detailed analysis of tachycardia in which multiple potentials (endocardial potential and far-field epicardial potential, etc) are recorded. In our case, even with the annotation-dependent EnSite X EP System, we were able to analyze the tachycardia mechanism in detail using OT, and by adjusting the window we were able to identify the entire circuit. We believe that the diagnosis of VOM-related AT using a high-resolution 3D mapping system allowed us to make the appropriate treatment choice of VOM ethanol infusion, and the treatment was successful.

## Conclusion

We report a case of MB-associated AT treated with the EnSite X EP System. In addition to the high-resolution performance of this system, it was also useful in assessing tachycardia by evaluating propagation with an annotation-dependent activation map and annotation-independent OT vectors, and by changing the roving activation intervals as needed for circuit identification.

## Appendix Supplementary data

Supplemental Figure 1 Fluoroscopic images during ethanol infusion and intracardiac ECG at the time of termination After confirming that the VOM was running near the left atrial appendage, ethanol injection was performed. AT was terminated about 2 seconds after injection of 2 mL ethanol 98%.

Supplemental Figure 2 Analysis of local potentials Comparing three types of potential at sites where epicardial potentials were recorded: the conventional bipolar potential, the best duplicate, which selects the higher potential in the direction along and across the spline; and the Omnipolar potential. The first component recorded with the Omnipolar was the most clear and the voltage was highest.
