# Supplementary figures and images for: Identifying the origin of atrial tachycardia in the epicardial region by analyzing two separate roving activation intervals using a novel three-dimensional mapping system: A case study
Source: HeartRhythm Case Rep. 2022 Jun 3;8(9):610–4. doi: 10.1016/j.hrcr.2022.05.024 (PMC9485655; doi:10.1016/j.hrcr.2022.05.024)

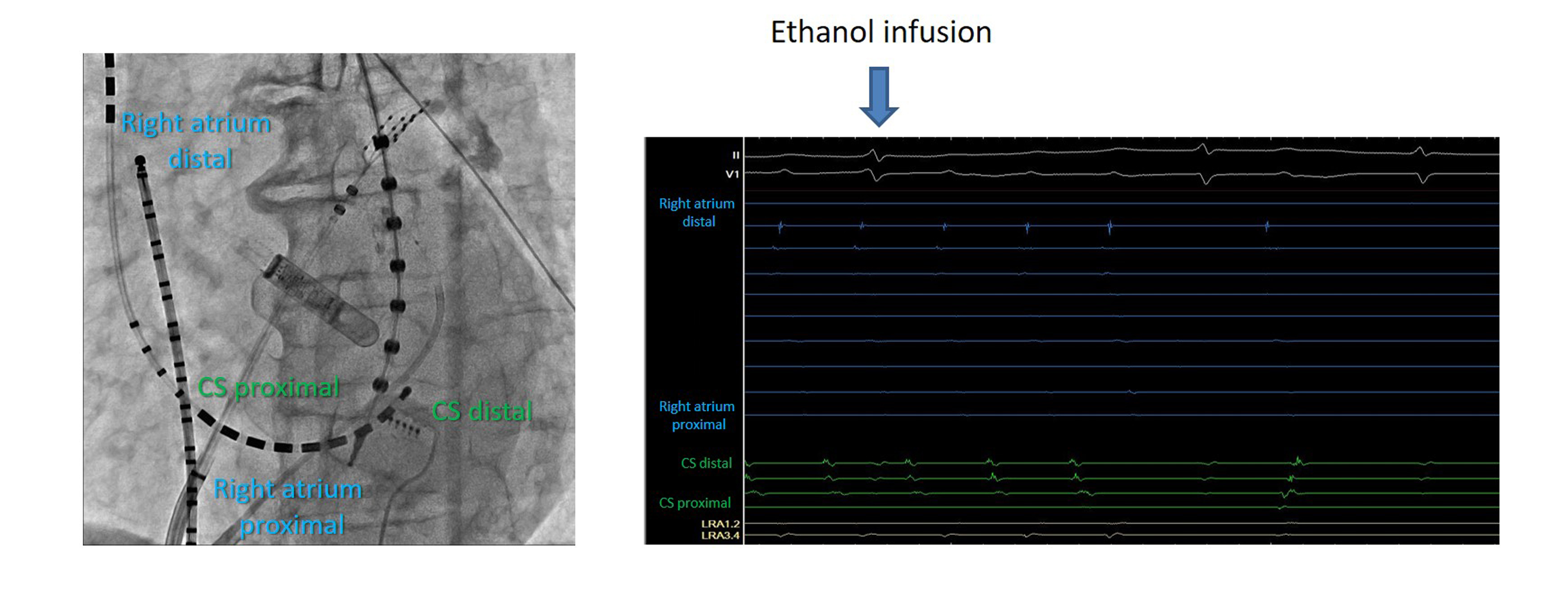

Supplement: Supplemental Figure 1 — Fluoroscopic images during ethanol infusion and intracardiac ECG at the time of termination After confirming that the VOM was running near the left atrial appendage, ethanol injection was performed. AT was terminated about 2 seconds after injection of 2 mL ethanol 98%. [file figs1.jpg]

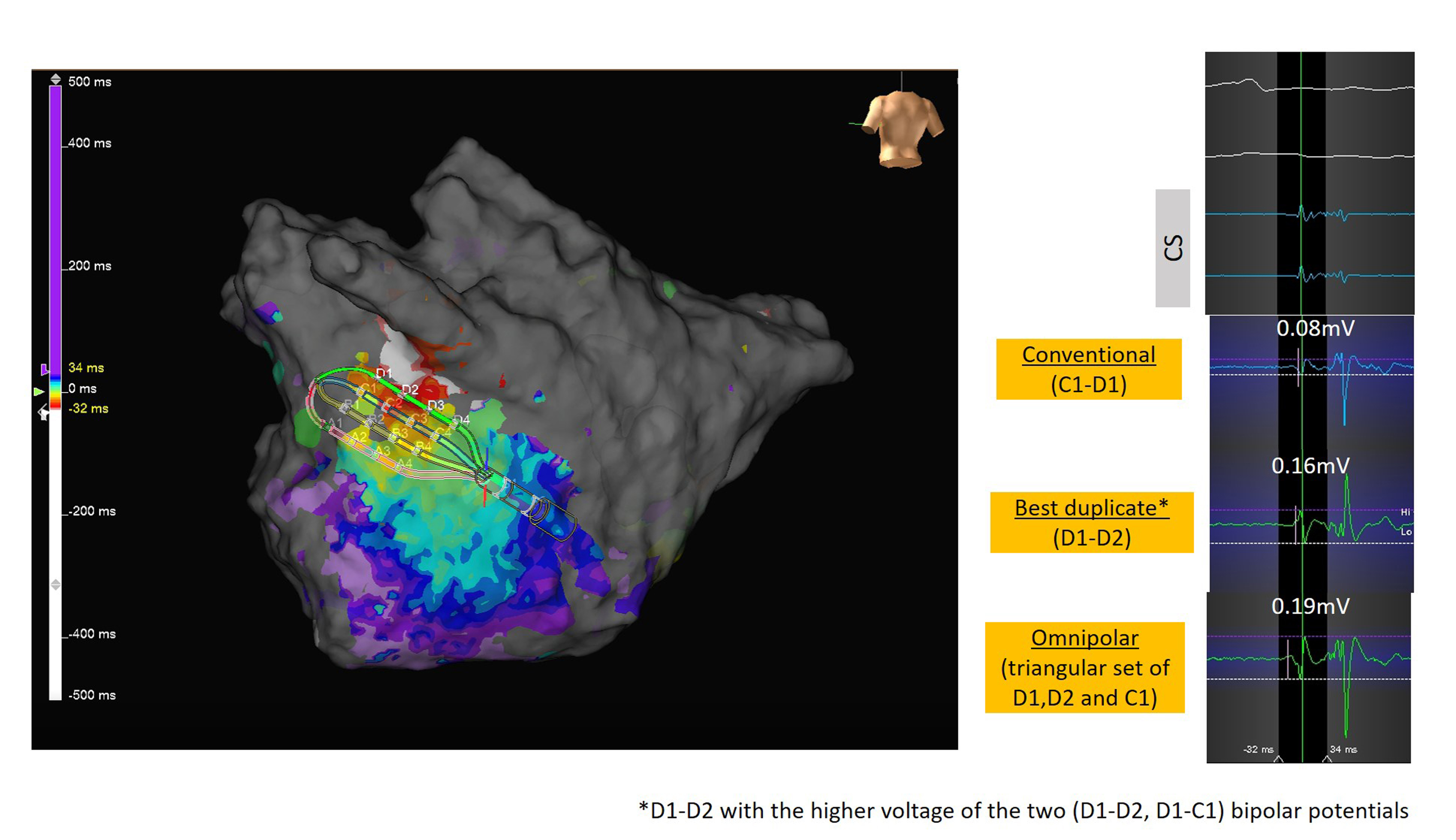

Supplement: Supplemental Figure 2 — Analysis of local potentials Comparing three types of potential at sites where epicardial potentials were recorded: the conventional bipolar potential, the best duplicate, which selects the higher potential in the direction along and across the spline; and the Omnipolar potential. The first component recorded with the Omnipolar was the most clear and the voltage was highest. [file figs2.jpg]
